# Maternal Cigarette Smoke Exposure Does Not Impair Influenza Vaccine Responsiveness in Murine Offspring

**DOI:** 10.3390/vaccines13101058

**Published:** 2025-10-16

**Authors:** Ali Dehghani, Johan Garssen, Ingrid van Ark, Gert Folkerts, Jeroen van Bergenhenegouwen, Saskia Braber

**Affiliations:** 1 Division of Pharmacology, Utrecht Institute for Pharmaceutical Sciences, Faculty of Science, Utrecht University, Universiteitsweg 99, 3584 CG Utrecht, The Netherlands; a.dehghani@uu.nl (A.D.); j.garssen@uu.nl (J.G.); i.vanark@uu.nl (I.v.A.); g.folkerts@uu.nl (G.F.); jeroen.vanbergen@danone.com (J.v.B.); 2 Outpatient Clinic for Occupational Medicine, Department of Public and Occupational Health, Amsterdam University of Medical Science, 1081 HV Amsterdam, The Netherlands; 3 Danone Research & Innovation, 3584 CT Utrecht, The Netherlands

**Keywords:** maternal exposure, air pollution, cigarette smoke, influenza vaccination, vaccine efficacy, early life

## Abstract

Background/Objectives: Environmental pollutants can profoundly affect immune development, yet their impact on offspring vaccine responsiveness remains poorly understood. To address this, we investigated the impact of maternal cigarette smoke (CS) exposure, a major contributor to household air pollution, on influenza vaccine responsiveness in offspring. Methods: Pregnant dams were exposed to CS or air during gestation and lactation. Two weeks post-weaning, offspring received two influenza vaccinations. After the booster vaccination, vaccine-specific delayed-type hypersensitivity (DTH), serum immunoglobulins, and splenic T cells were analyzed. Results: Vaccinated offspring exhibited robust DTH responses and comparable levels of vaccine-specific IgG1 and IgG2a, regardless of maternal exposure. Importantly, maternal CS exposure did not affect splenic Th1 cell frequency in vaccinated offspring but increased the frequency of activated Th2 cells. Conclusions: In conclusion, immune development was affected by enhanced Th2 activation, but vaccine efficacy was not impaired. These findings suggest that, under the current conditions of CS exposure (duration, route, and timing) and influenza vaccine dose, vaccine-induced immunity may exhibit resilience even in the presence of environmental immune modulators such as maternal CS exposure. However, these unexpected results highlight the need for further investigation into the broader health implications of maternal pollutant exposure, particularly considering how exposure timing, type, and route, as well as vaccine characteristics, may influence immune development and responsiveness. A deeper understanding of these factors is essential to fully elucidating the clinical relevance of maternal pollutant exposure on childhood vaccine efficacy.

## 1. Introduction

Children in low- and middle-income countries face significant risks from household air pollution generated by cooking and heating fuels, as well as environmental cigarette smoke [1,2]. This exposure is particularly concerning for pregnant women and their unborn babies, as prenatal exposure to household air pollution can lead to long-term health issues, including impaired immune development in mothers [3] and offspring [4,5,6]. Children are particularly vulnerable to the adverse effects of pollutants and viral infections such as influenza due to their developing lungs and immature immune systems [7]. Infants of smokers have much higher rates of respiratory infection [6], and annually, approximately 570,000 children under the age of five die from respiratory infections linked to indoor and outdoor air pollution [8]. Influenza is a major contributor of acute lower respiratory infections in children, particularly in low- and lower-middle-income countries. In 2018, the influenza virus was estimated to be responsible for approximately 10 million acute lower respiratory infection cases and 870,000 hospitalizations in the under-five pediatric population. Infants younger than six months were especially vulnerable, accounting for 23% of hospital admissions and 36% of in-hospital deaths related to influenza [9]. These statistics underscore the urgent need to investigate the impact of air pollution on respiratory health and infection in children [10].

Childhood vaccinations play a crucial role in protecting public health and preventing severe infections. However, vaccine efficacy depends on a healthy immune system, which can be compromised by environmental pollution. A systematic review of clinical and observational data demonstrated that pollution can adversely affect immune responses to vaccines [11]. Several in vivo and in vitro studies have shown that CS can weaken the innate antiviral response to influenza [12,13,14,15].

Cohort studies [16,17] have shown that air pollution exacerbates chronic lung and systemic inflammation, potentially impairing vaccine efficacy in the general population [18], healthcare workers [19], and children [20], similar to its effects in other chronic inflammatory conditions such as pneumonia in children [21].

The complex interaction between household air pollution, such as CS, and the immune system is often mediated via inflammation. These inflammatory processes can influence the immune response to vaccination. Direct effects of pollutants on vaccine efficacy have been documented in some in vivo and clinical studies [22,23,24]. Interestingly, the relationship between air pollution and the immune system may not be entirely unidirectional. For example, the influenza vaccine has been shown to moderate the harmful effects of ambient air pollution on lung function in children, suggesting that vaccination may also confer some protective benefits against pollution [25,26]. This bidirectional relationship underscores the need to explore how pollution impacts vaccination outcomes and vice versa.

Understanding how maternal pollutant exposure alters offspring immune responses is essential for informing clinical vaccination strategies and optimizing immunization outcomes in early life [27,28]. However, it remains unclear whether prenatal and postnatal exposure to air pollutants, particularly maternal CS, dampens or modifies vaccine-induced immunity. Some studies have reported impaired immune responses; for example, maternal exposure to consumer product pollutants, such as per- and polyfluoroalkyl substances, has been associated with reduced immune responses to the measles vaccine in children [27]. In addition, maternal smoking during pregnancy has been shown to negatively affect the offspring’s immune system, contributing to neurobehavioral and neurodevelopmental complications [29], childhood asthma and allergic rhinitis [30], as well as increased lung Th2 cell frequencies and elevated serum levels of house dust mite (HDM)-specific IgE and IgG1 in HDM-treated offspring compared to those born to air-exposed mothers [31]. Although these studies did not directly assess vaccine efficacy, they suggest a heightened immunological vulnerability in offspring exposed to maternal pollutants and underscore the need to investigate whether such immune alterations lead to diminished vaccine responsiveness.

Building on our previous findings that offspring of maternal CS-exposed mice exhibit an enhanced Th2 response and greater susceptibility to airway allergy following HDM exposure [32], this study investigates the interplay between prenatal and postnatal environmental exposures and immune responses to vaccination. Specifically, we aim to assess the efficacy of influenza vaccination in the offspring of dams exposed to CS, using an established murine vaccination model from our group [33,34]. For the first time, we evaluate known vaccination responses—including both humoral and cellular immunity—with a specific focus on DTH responsiveness, vaccine-specific immunoglobulins, and splenic T cell subsets. These are assessed under a defined maternal CS exposure protocol in terms of duration, timing (gestation and lactation), route (inhalation), and a standardized vaccination schedule and dose, to understand how maternal CS exposure influences vaccination effectiveness.

## 2. Materials and Methods

### 2.1. Animals

A total of 63 BALB/cByJ mice (42 females and 21 males), aged eight weeks, were purchased from Charles River Laboratories (Charles River Laboratories, Den Bosch, The Netherlands). All animals were housed in an isolated room within the animal facility under standardized conditions, including a regulated ambient temperature of 21 ± 2 °C, relative humidity of 50–55%, and a 12-h light/dark cycle (lights on from 7:00 a.m. to 7:00 p.m.). Enrichment materials included wood-chip bedding (Tecnilab-BMI, Someren, The Netherlands), plastic shelters (Animalab, Poznan, Poland), and tissues (VWR, Amsterdam, The Netherlands) provided within the cages. Throughout the study, mice were given unrestricted access to tap water and a standard rodent diet (AIN-93G, Ssniff Spezialdiäten, Soest, Germany). After being transferred to the animal facility, mice underwent a 2-week acclimatization period, during which they were group-housed in static microisolator cages (filter-topped Makrolon^®^, 22 cm × 16 cm × 14 cm, Tecnilab-BMI, Someren, The Netherlands). The mice were housed by sex, with six females or five males per cage.

These experiments comply with the ARRIVE guidelines and were conducted in accordance with the institutional policies of Utrecht University. All animal procedures were approved by the local Animal Welfare Body under ethical license AVD1080020184724, granted by the national competent authority (Centrale Commissie Dierproeven, CCD), ensuring full compliance with the European Directive 2010/63/EU.

### 2.2. Study Design

Using a random number generator, two female mice were caged following acclimatization and assigned to either the air or CS exposure groups. For mating, one male was placed in each cage with two females for a period of four days. After this mating period, the male mice were removed from the cages and excluded from the experiment. Whole-body exposure to air or cigarette smoke was administered to female mice from post-mating until the end of lactation. During the lactation period, offspring remained in the home cage with additional tissues provided to help maintain body temperature.

After mating, the two mothers and their respective offspring were housed together in cages and provided with a standard AIN-93G diet until the end of the lactation period.

On postnatal day 21, pups were weaned, and their sex was identified to help maintain balanced group compositions. Offspring were then pooled based on maternal exposure (air or CS) and randomly assigned to experimental groups to reduce potential litter bias. Despite this effort, a possible limitation of the study is the uneven representation of pups from the same litter within certain groups. The offspring were allocated into four subgroups based on subcutaneous injections of either undiluted Influvac or phosphate-buffered saline (PBS). Male and female offspring were housed separately, with 3 to 5 offspring per cage. To minimize potential confounders, treatments and measurements were randomized across litters, and cage positions were rotated regularly in the isolated room.

### 2.3. CS Exposure Procedure

Female mice were gradually acclimated to CS by increasing the number of 1R6F reference cigarettes (Kentucky, Lexington, KY, USA) over the first week, reaching 14 cigarettes/day by day 7 (approx. 50 min/day), as previously described [32]. This exposure continued daily for 6 weeks, from gestational day 4 to the end of lactation. During the exposure period, smoke levels were monitored daily by measuring the carbon monoxide and total particulate matter concentrations. Total particulate matter levels were measured using gravimetric analysis with a type A/E glass fiber filter (PALL Life Sciences, Morelos, Mexico). Total particulate matter (TPM) concentrations began at around 237 μg/L and gradually increased to approximately 828 μg/L when 14 cigarettes were used. Carbon monoxide was consistently maintained between 300 and 400 ppm [32].

### 2.4. Vaccination Model

**Figure 1:**
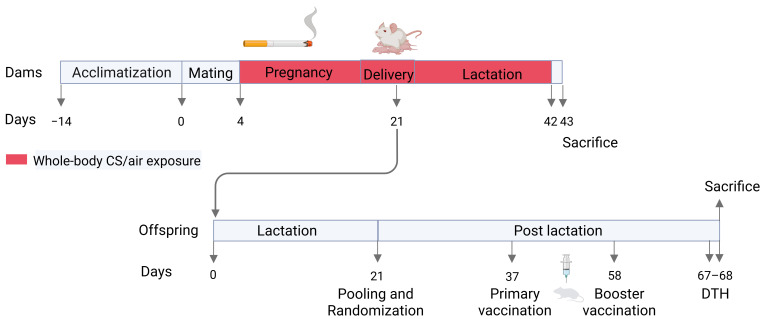
Experimental design and animal groups. Offspring were exposed to air or CS during both the prenatal and postnatal periods and received primary and booster subcutaneous injections of either Influvac or PBS (control). DTH: delayed-type hypersensitivity.

On postnatal day 37, offspring from both air- and CS-exposed dams received a primary subcutaneous injection of 125 μL Influvac (Abbott Biologicals B.V., Weesp, The Netherlands). This inactivated influenza vaccine (2019/2020 formulation) contains hemagglutinin (HA) and neuraminidase antigens from three influenza virus strains, with a total HA concentration of 90 μg/mL. The booster was administered three weeks after the primary vaccination. Control group offspring from both exposure groups were injected subcutaneously with 125 μL of PBS. Delayed-type hypersensitivity to Influvac was induced on postnatal day 67, nine days after the booster, and ear swelling was evaluated 24 h later under deep isoflurane anesthesia [35]. A schematic overview of the study design is shown in Figure 1.

### 2.5. Antigen-Specific Delayed-Type Hypersensitivity (DTH) Reaction

DTH reaction was measured using a previously described method [33]. To induce the response, 20 μL of Influvac was injected intradermally into the right ear pinna, while the left ear received the same amount of PBS to account for nonspecific swelling. Ear thickness was measured using a digital micrometer (Mitutoyo Digimatic) at two time points: baseline (0 h, day 67) and 24 h later (day 68). The DTH response was calculated as ΔDTH (µm) = ear thickness at 24 h − ear thickness at 0 h. Antigen-specific responses were determined as ΔΔDTH (µm) = ΔDTH of the right ear − ΔDTH of the left ear. To ensure animal welfare, mice were closely monitored for signs of discomfort or distress following intradermal injection. On postnatal day 68, twenty-four hours after the intradermal ear challenge, blood samples were collected via orbital sinus under isoflurane anesthesia. Finally, the offspring were euthanized via cervical dislocation, and samples, including blood and spleen, were collected for further analyses.

### 2.6. Influvac-Specific Antibody Analysis in Serum

Offspring blood samples were centrifuged at 12,000 RCF for 10 min to separate the serum, which was subsequently stored at −20 °C. Serum levels of Influvac-specific immunoglobulin (Ig)G1 and IgG2a were quantified using an enzyme-linked immunosorbent assay (ELISA) as previously described [33].

### 2.7. Flow Cytometric Analysis of Spleen

T-cell populations were quantified in spleen samples using flow cytometry [33]. Splenocytes were isolated by mechanically dissociating spleen tissue through a nylon mesh cell strainer (Falcon; Becton Dickinson, Vianen, The Netherlands), followed by red blood cell lysis using a commercial lysis buffer (Thermo Scientific Chemicals, USA). The lysis reaction was quenched by adding 5 mL of RPMI supplemented with 10% fetal calf serum (FCS) and 1% penicillin/streptomycin (Pen/Strep). After a second centrifugation at 1400 rpm for 5 min at 4 °C, the supernatant was discarded, and the cell pellet resuspended in 2 mL of RPMI-10% FCS-1% Pen/Strep medium. For cell counting, 25 μL of the suspension was diluted in 10 mL of isotonic solution (1:400 dilution) and counted using a Coulter counter. After counting, the cells were incubated at room temperature for 1 h and subsequently stained for surface markers, including CD4 Brilliant Violet 510, CCR6-PE (BioLegend, San Diego, CA, USA), CD69-PE-Cy7, CXCR3-PE, CD25-PerCP-Cy5.5, (eBiosciences, Thermo Fisher Scientific, San Diego, CA, USA), and T1/ST2-FITC (MD Biosciences, St. Paul, MN, USA). Cell viability was assessed using the fixable viability dye eFluor^®^ 780 (eBioscience, San Diego, CA, USA). Cells were fixed and permeabilized using a fixation/permeabilization buffer set (eBioscience, San Diego, CA, USA) prior to intracellular staining with Foxp3-FITC, GATA3- PerCP-eFluor710, RORγT-PE (eBiosciences, San Diego, CA, USA), and Tbet- Alexa Fluor647 (BioLegend, San Diego, CA, USA) antibodies. Data acquisition was performed using a BD FACSCanto II flow cytometer (Becton Dickinson, Franklin Lakes, NJ, USA), and analysis was conducted with FlowLogic software, FlowLogic Solution 1.3 (Inivai Technologies, Mentone, VIC, Australia).

### 2.8. Statistical Analysis

Statistical tests were carried out using GraphPad Prism, version 10 (San Diego, CA, USA). The sample size calculation was based on the DTH response, the key outcome parameter in the influenza vaccination model. For model validation purposes, PBS-treated offspring maternally exposed to air were included as a reference group. In this context, a group size of n = 3 was deemed sufficient to establish a stable baseline response, as supported by previous studies [33]. Data were analyzed using two-way ANOVA (with treatment (CS vs. air), and vaccination (PBS vs. influenza vaccine) as factors), followed by Bonferroni’s post hoc multiple comparisons test with a *p*-value of <0.05. Sex-specific analyses were limited due to small numbers in some subgroups. For comparisons between two groups, Student’s *t*-test was applied. The data are presented as mean ± standard error of the mean (SEM).

## 3. Results

### 3.1. Effects of Maternal CS Exposure on Birth Outcomes and Spleen-to-Body Weight Ratio in Offspring

A total of 42 female mice were included in the study, with 16 assigned to the air-exposed group and 26 to the CS-exposed group. Of these, 6 air-exposed and 7 CS-exposed females became pregnant, while the remaining mice were non-pregnant (air group: 10; CS group: 19). These pregnancies resulted in 47 offspring in total: 18 from air-exposed dams (7 males, 11 females) and 29 from CS-exposed dams (9 males, 20 females). Since mating was conducted over a 4-day window, the precise timing of fertilization could not be determined for each dam, potentially contributing to variation in gestational length. For consistency in data interpretation, the first day of the mating period was uniformly considered as gestational day one for all animals, as detailed in Table 1. CS exposure during pregnancy and lactation did not affect the duration of pregnancy, litter size, or sex ratio of the offspring between groups. The body weight of 1-week-old offspring born to CS-exposed dams was significantly lower than that of offspring born to air-exposed dams. Ten-week-old male offspring of CS-exposed dams exhibited a significantly higher spleen-to-body weight ratio compared to those from the air-exposed group (Table 1).

### 3.2. Comparable DTH Response and Serum IgG1 and IgG2a Levels in Vaccinated Offspring of Air- or CS-Exposed Dams

**Figure 2:**
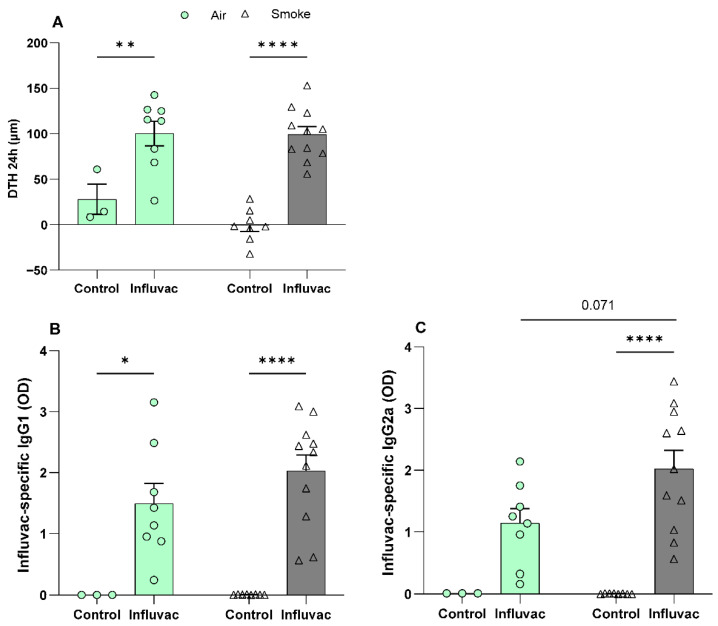
Comparable DTH response and serum IgG1 and IgG2a levels in vaccinated offspring of air- or CS-exposed dams. Offspring were exposed to either air or CS both prenatally and postnatally. After weaning, offspring received primary and booster subcutaneous injections of either PBS (control) or Influvac. Ten days after the second injection, Influvac-specific DTH (**A**) was assessed by measuring ear thickness changes 24 h after intradermal challenge with Influvac. Influvac-specific IgG1 (**B**) and IgG2a (**C**) levels were measured in serum. Air–PBS, n = 3; Air–Influvac, n = 8; Smoke–PBS, n = 8; Smoke–Influvac, n = 11. Data are expressed as mean ± SEM. Statistical significance was determined using two-way ANOVA: * *p* < 0.05, ** *p* < 0.01, and **** *p* < 0.0001.

Influenza vaccination efficacy was evaluated by assessing both local and systemic responses. Maternal CS exposure did not significantly affect the DTH response in the ears of vaccinated offspring compared to those maternally exposed to air (Figure 2A).

The humoral immune response was assessed by measuring IgG antibody levels. Results from the Influvac vaccination-induced model indicate that all vaccinated offspring had significantly higher levels of Influvac-specific IgG1 and IgG2a compared to the control group (PBS-injected) (Figure 2B,C), except for IgG2a levels in air-exposed vaccinated offspring, which showed a tendency to increase rather than reaching statistical significance (Figure 2C). However, maternal CS exposure did not affect the serum IgG1 and IgG2a levels in vaccinated offspring compared to those born to air-exposed mothers (Figure 2B,C), although a trend towards a difference in IgG2a levels was observed. In addition, this pilot study included small dietary intervention groups to explore potential factors affecting vaccine responses (air-exposed + Influvac + diet: n = 7; CS-exposed + Influvac + diet: n = 10). As maternal CS exposure did not significantly affect vaccine outcomes, these exploratory groups were excluded from the present analysis to maintain focus on the main study objectives.

### 3.3. Maternal CS Exposure Does Not Affect Splenic Th1 Infiltration or Activation but Increases the Frequency of Activated Th2 Cells in Offspring

**Figure 3:**
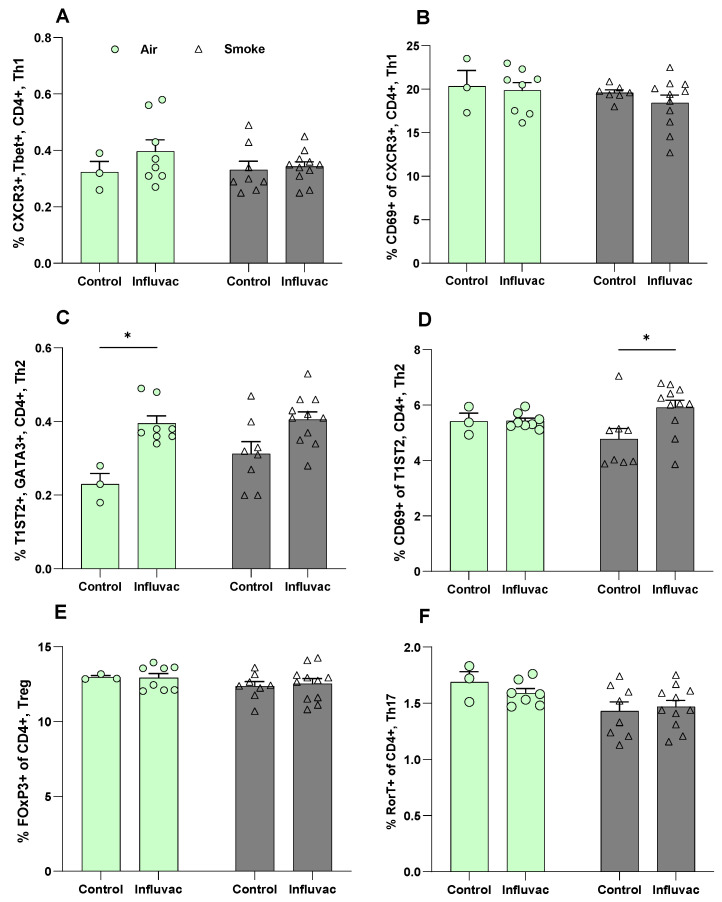
Maternal CS exposure does not affect splenic Th1 infiltration or activation but increases the frequency of activated Th2 cells in offspring. Offspring were exposed to either air or CS both prenatally and postnatally. After weaning, offspring received primary and booster subcutaneous injections of either PBS (control) or Influvac. Ten days after the booster vaccination, offspring were euthanized, and spleen tissues were collected for splenocyte isolation and subsequent quantification of T cell subsets. Percentages of (**A**) Th1 cells (% CXCR3+, Tbet+, CD4+), (**B**) activated Th1 cells (% CD69+ of CXCR3+, CD4+), (**C**) Th2 cells (% T1ST2+, GATA3+, CD4+), (**D**) activated Th2 cells (% CD69+ of T1ST2+, CD4+), (**E**) Treg cells (% FoxP3+ of CD4+), and (**F**) Th17 cells (% RORγT+ of CD4+) are shown. Air–PBS, n = 3; Air–Influvac, n = 8; Smoke–PBS, n = 8; Smoke–Influvac, n = 11. Data are presented as mean ± SEM; * *p* < 0.05, as determined by two-way ANOVA.

Cellular immune responses were assessed by measuring the frequencies of Th1, Th2, Treg, and Th17 cells in spleen samples from vaccinated offspring using flow cytometry (Figure 3A–F), following the gating strategies shown in Supplementary Figures S1 and S2. Influvac did not affect the frequency or activation of Th1 cells in offspring born to either air- or CS-exposed dams (Figure 3A,B). However, Influvac resulted in an increased frequency of Th2 cells and activated Th2 cells in offspring born to air-exposed and CS-exposed dams, respectively (Figure 3C,D). Additionally, Influvac did not affect the frequencies of Treg and Th17 cells in offspring from either air- or CS-exposed dams (Figure 3E,F).

## 4. Discussion

In the present study, we employed a preclinical murine influenza virus vaccination model to investigate, for the first time, the potential detrimental effects of maternal CS exposure on the vaccination response in offspring. Environmental cigarette smoke is a major source of indoor air pollution and is considered one of the leading health risk factors due to its potent immunotoxic effects [1]. Early life exposure to air pollution has been shown to impair immune development [1,2,36], particularly by disrupting the Th1/Th2 balance in offspring, often skewing immunity towards a Th2 phenotype and increasing susceptibility to allergens [32,37,38,39].

Vaccination is a well-established method to evaluate host immune function, with serum antigen-specific IgG1 and IgG2a antibodies serving as markers for Th2 and Th1 responses, respectively [40,41]. While early-life vaccination is critical for establishing robust immune protection, infants naturally display a Th2-biased immune response [42]. This innate bias may be exacerbated by maternal exposure to air pollutants, potentially further favoring Th2 over Th1 immunity [1,2]. Given this background, we hypothesized that maternal CS exposure would impair vaccine efficacy in offspring by suppressing Th1 responses, underscoring the need to deepen our understanding of immune regulation during vaccination. Our previous data suggest that elevated epidermal growth factor (EGF) levels in smoke-exposed dams and their offspring [43] may contribute to immune dysregulation, disrupting the Th2/Th1 balance [44] and potentially compromising vaccine responses.

The physiological effects of maternal CS exposure were evident in both body weight and the spleen-to-body weight ratio of offspring. Reduced body weight following maternal CS exposure may result from smoke components crossing the placenta and impairing fetal growth [45]. In addition, male offspring from mothers exposed to CS showed an increased spleen-to-body weight ratio compared with those from mothers exposed to air suggesting spleen enlargement, which may reflect immune system activation or an inflammatory response. Prenatal and postnatal CS exposure is known to induce systemic inflammation and immune modulation, which may lead to alterations in the size of immune-related tissues, such as the spleen. Supporting this, both maternal and paternal smoking have been associated with alterations in spleen weight and spleen-to-body weight ratios in offspring postnatally, potentially mediated by epigenetic modifications or hormonal imbalances [43,46]. These mechanisms may contribute to the observed sex-specific differences, as male and female offspring can respond differently to environmental exposures due to variations in sex hormones and immune development. Nonetheless, the precise pathways underlying these effects remain unclear and warrant further investigation.

In our study, the kinetics of immune responses to vaccination were reflected by significantly increased DTH responses and vaccine-specific serum IgG2a and IgG1 levels, consistent with findings from other murine studies [33,34]. However, no differences were observed in splenic Th1 cell frequency. The observed antibody production despite unaltered Th1 cell frequency may reflect vaccine-induced immune activation mechanisms. Vaccines can stimulate antibody production via both T-cell-dependent and T-cell-independent pathways [33,47]. T-cell-independent antigens, for instance, activate B cells directly through B-cell receptors (BCRs) and toll-like receptors (TLRs), bypassing the need for CD4+ Th cell involvement [47,48,49].

Contrary to our initial hypothesis, vaccinated offspring from both air- and CS-exposed dams showed comparable DTH responses and vaccine-specific antibodies, suggesting that maternal CS exposure did not impair overall vaccine efficacy in this model. Moreover, the percentage of splenic Th1 cells and activated Th1 cells were unaffected, aligning with previous studies on maternal exposure to low-dose particulate matter that showed limited impact on splenic T-bet expression or CD4+ T cell populations [50,51]. However, there was some indication that vaccination may enhance the frequency of activated Th2 cells in the spleens of CS-exposed offspring. Interestingly, in contrast to our findings, a previous study from our group demonstrated that maternal exposure to deoxynivalenol, a food contaminant, during pregnancy significantly reduced vaccine responsiveness in offspring. This reduction was evident through decreased DTH responses, lower serum levels of vaccine-specific IgGs, reduced percentages of T-bet+ Th1 cells, and diminished production of Th1 cytokines, including IFN-γ, IL-12p70, and IL-27. However, no significant differences were observed in Th2 or CXCR3+ Th1 cell populations [33]. This disparity may be attributed to differences in exposure routes and pollutant types. Additionally, air pollution has been shown to induce mixed Th1/Th2 inflammatory responses in both in vivo [52] or human [53] studies, with variations in findings likely due to differences in PM composition, exposure duration, and study protocols. Such inconsistencies highlight the complexity of air pollution’s immunological effects and suggest that different pollutants may exert distinct influences on immune programming. Future studies should further explore these pollutant-specific effects, including potential interactions with vaccination, to better understand how environmental exposures shape early-life immune development.

The increased frequency of activated Th2 cells observed in our study may reflect an underlying predisposition toward Th2 polarization, consistent with previous reports linking maternal CS exposure to a Th2-biased immune profile in offspring [32,54,55] similar to findings in infected mice at the onset of patency [56] and in genetically Th2-prone mouse strains, such as BALB/c [57,58]. This predisposition could influence vaccine-induced immunity, shifting the response towards Th2 rather than Th1. Future studies using Th1-biased strains, such as C57BL/6, could help determine whether the effects of CS exposure and subsequent immune polarization are strain-dependent or represent a broader immunological phenomenon. Although this Th2-skewed response did not appear to impair overall vaccine efficacy in our study, it raises important clinical questions regarding long-term immune resilience, particularly against intracellular pathogens that require strong Th1-mediated immunity.

Epidemiological studies have linked maternal exposure to pollutants such as per- and polyfluoroalkyl substances, commonly found in consumer products, to diminished immune responses to vaccines, including lower IgG reactivity to measles vaccine in children [27,59]. Additionally, a study on direct adult exposure to perfluorooctanoate showed that elevated serum perfluorooctanoate concentrations were associated with a lower rise in antibody titers, particularly against the A/H3N2 influenza virus, and an increased risk of not reaching the antibody threshold required for long-term protection [60]. The discrepancy between these findings and our study may be due to differences in pollutant type, route of exposure, or the life stage at which exposure occurred. Similarly, both clinical [29] and preclinical [31] evidence have demonstrated that maternal smoking during pregnancy negatively affects the offspring’s immune system, leading to neurobehavioral and neurodevelopmental complications [29] and increased lung Th2 cell frequencies, elevated serum levels of HDM-specific IgE and IgG1, and reduced lung Th1 cell frequencies [31]. Importantly, while these studies clearly demonstrate immunological alterations, they do not assess whether these effects are sufficient to impair vaccine responsiveness. Our study specifically addresses this gap by evaluating vaccine-induced immune responses under a defined CS exposure protocol. This observation does not negate the immunotoxic effects of maternal CS exposure but instead highlights a potentially preserved systemic immune capacity under certain exposure and vaccination conditions. Notably, our findings suggest a divergence between local and systemic T cells responses: whereas maternal smoking is known to impair lung immunity, systemic vaccine responses, such as splenic Th1, appeared preserved in our model. These differences may stem from tissue-specific immune reprogramming, where localized effects in the lungs [32] differ from systemic responses in compartments such as the spleen [31,50,51]. This nuance adds valuable context to the existing literature and underscores the need for further investigation into the broader health implications of maternal pollutant exposure. Future studies should consider the type, route, timing, and duration of exposure, as well as the distinction between localized and systemic immune effects, when evaluating vaccine-induced immune responses.

The absence of an observed detrimental effect of maternal CS exposure on vaccination outcomes in our model may be explained by the robust nature of vaccine-induced immunity. The strong immune stimulation elicited by the influenza vaccine may have overridden subtle immunotoxic effects of CS exposure under the current conditions. Alternatively, the relatively long and consistent CS exposure might have induced immune changes while still preserving vaccine responses. Future studies employing suboptimal vaccination models might better reveal subtle immunomodulatory effects of maternal CS exposure.

Limitations of our study include the timing of exposure, which began at gestational day 4, whereas real-life maternal exposures often commence prior to conception and continue postpartum. While we acknowledge this discrepancy, pre-mating CS exposure could have impaired fertility and resulted in insufficient litters for follow-up studies. Any observed effects in the offspring could result from in utero exposure, postnatal exposure through lactation, or a combination of both. While we acknowledge this as a limitation, future studies aimed at isolating these exposure windows, such as exposing dams only during gestation or only during lactation, would be valuable for elucidating the relative contributions of prenatal versus postnatal CS exposure on offspring outcomes. Moreover, our study builds on previous work using the same design to investigate maternal CS exposure and Th2/Th1 responses. Despite these limitations, the findings offer biological plausibility supporting epidemiological evidence that maternal smoking or exposure to household air pollution increases the risk of allergic diseases in offspring [32]. Further research investigating other immune compartments or functional assays (e.g., lung-resident cells or cytokine profiling) is needed to provide additional insight into the mechanisms underlying CS-induced modulation of vaccine responses and to deepen our understanding of these effects. Finally, sex-specific differences were not analyzed, as the primary objective of this study was to evaluate the effects of maternal CS exposure on vaccine efficacy in offspring. The study’s power calculation was based on offspring randomization without sex stratification to ensure sufficient sample size for the primary outcome. Consequently, potential sex-related variations were not accounted for, and sex-specific analyses were limited by the small numbers in some subgroups. Future studies with larger sample sizes should explore these sex-specific differences to provide a more comprehensive understanding of vaccine efficacy.

## 5. Conclusions

In summary, our findings indicate that vaccinations influenced most measures of the immune system in this study, while maternal CS exposure had only limited effects. Vaccine-induced humoral immunity appeared to be preserved under the current conditions of maternal CS exposure, as reflected by intact DTH responses and vaccine-specific IgG1 and IgG2a production. These results were somewhat unexpected given earlier reports of heightened allergic sensitivity in CS-exposed offspring, highlighting the complexity of early-life immune modulation. While our data indicate a degree of resilience in systemic vaccine responses, these findings should be interpreted with caution. They are specific to the exposure protocol and vaccine used in this study and may not be generalizable to other pollutants, exposure conditions, or immunization strategies. Importantly, our results should be viewed as an initial investigation that offers complementary insights into how maternal CS exposure may or may not affect vaccine-induced immunity. Future research is needed to investigate additional variables that may influence immune development and vaccine responsiveness, including pollutant type, exposure duration and timing, exposure route, sex-specific differences, other immune compartments, and different mouse strains. Moreover, evaluating other vaccine types and models with lower immunogenicity may uncover immunosuppressive effects that were not detectable in the present study.

## Figures and Tables

**Table 1:** Effects of CS exposure on birth outcomes and spleen-to-body weight ratio.

	Experimental Groups		*p* Value
	Air	CS	
Duration of pregnancy ^a^	22.00 ± 0.2	22.43 ± 0.25	
Litter size (median)	4	3.5	
Number of pregnant dams ^b^	6	7	
Female/male ratio	1.57	2.22	
Body weight offspring (1 week old) ^c^	5.45 ± 0.14	4.48 ± 0.1 ****	0.0001
Spleen weight/body weight ratio			
Female	0.005 ± 0.0006	0.005 ± 0.0006	
Male	0.0033 ± 0.0001	0.0038 ± 0.0001 *	0.015

^a^ The first day of the 4-day mating period was designated as gestational day 1. ^b^ Values indicate the number of pregnant dams per group (6 pregnant dams from a total of n = 16 for the air group and 7 pregnant dams from a total of n = 26 for the CS group). ^c^ Statistical differences were evaluated using Student’s *t*-test, with * *p* < 0.05 and **** *p* < 0.0001 considered significant compared to offspring from air controls. Results: mean ± SEM.

## Data Availability

The original contributions presented in the study are included in the article/Supplementary Materials. Further inquiries can be directed to the corresponding author.

## Supplementary Materials

The following supporting information can be downloaded at: https://www.mdpi.com/article/10.3390/vaccines13101058/s1, Supplementary Figure S1. Gating strategy used for FACS analysis of T cells in solenocytes from Influenza-vaccinated offspring born to air-exposed and CS-exposed dams, performed using FlowLogic software. Supplementary Figure S2. Gating strategy used for FACS analysis of Th2 cells (% T1ST2+, GATA3+, CD4+) in solenocytes from Influenza-vaccinated offspring in offspring born to air-exposed and CS-exposed dams, performed using FlowLogic software.

## References

[1] Saxena K. (2024). Association Between Maternal Prenatal Exposure to Household Air Pollution and Child Respiratory Health: A Systematic Review and Meta-analysis. Yale J. Biol. Med..

[2] Liu K., Zhang H., Bo Y., Chen Y., Zhang P., Huang C., Yu Z., Gao Z. (2024). Ambient air pollution and Children’s health: An umbrella review. Atmos. Pollut. Res..

[3] Drury N.L., Mustapha T., Shore R.A., Zhao J., Wright G.A., Hoffmann A.R., Talcott S.U., Regan A., Tighe R.M., Zhang R. (2023). Maternal exposure to ultrafine particles enhances influenza infection during pregnancy. Part. Fibre Toxicol..

[4] Lin L.Z., Chen J.H., Yu Y.J., Dong G.H. (2023). Ambient air pollution and infant health: A narrative review. EBioMedicine.

[5] Hehua Z., Qing C., Shanyan G., Qijun W., Yuhong Z. (2017). The impact of prenatal exposure to air pollution on childhood wheezing and asthma: A systematic review. Environ. Res..

[6] Metzger M.J., Halperin A.C., Manhart L.E., Hawes S.E. (2013). Association of maternal smoking during pregnancy with infant hospitalization and mortality due to infectious diseases. Pediatr. Infect. Dis. J..

[7] Yaremenko A.V., Pechnikova N.A., Porpodis K., Damdoumis S., Aggeli A., Theodora P., Domvri K. (2024). Association of Fetal Lung Development Disorders with Adult Diseases: A Comprehensive Review. J. Pers. Med..

[8] WHO The Cost of a Polluted Environment: 1.7 Million Child Deaths a Year, Says WHO. https://www.who.int/news/item/06-03-2017-the-cost-of-a-polluted-environment-1-7-million-child-deaths-a-year-says-who.

[9] Wang X., Li Y., O’Brien K.L., Madhi S.A., Widdowson M.A., Byass P., Omer S.B., Abbas Q., Ali A., Amu A. (2020). Global burden of respiratory infections associated with seasonal influenza in children under 5 years in 2018: A systematic review and modelling study. Lancet Glob. Health.

[10] Kielsen K., Shamim Z., Ryder L.P., Grandjean P., Heilmann C., Esser C. (2016). Vaccination Efficacy and Environmental Pollution. Environmental Influences on the Immune System.

[11] Protano C., Valeriani F., Vitale K., Del Prete J., Liguori F., Liguori G., Gallè F. (2024). Exposure to Pollutants and Vaccines’ Effectiveness: A Systematic Review. Vaccines.

[12] Wu W., Zhang W., More S., Booth J.L., Duggan E.S., Liu L., Zhao Y.D., Metcalf J.P. (2014). Cigarette smoke attenuates the RIG-I-initiated innate antiviral response to influenza infection in two murine models. Am. J. Physiol. Lung Cell Mol. Physiol..

[13] Wu W., Alexander J.S., Metcalf J.P. (2022). In Vivo and In Vitro Studies of Cigarette Smoke Effects on Innate Responses to Influenza Virus: A Matter of Models?. Viruses.

[14] Chavez J., Hai R. (2021). Effects of Cigarette Smoking on Influenza Virus/Host Interplay. Pathogens.

[15] Danov O., Wolff M., Bartel S., Böhlen S., Obernolte H., Wronski S., Jonigk D., Hammer B., Kovacevic D., Reuter S. (2020). Cigarette Smoke Affects Dendritic Cell Populations, Epithelial Barrier Function, and the Immune Response to Viral Infection With H1N1. Front. Med..

[16] Tripathy S., Marsland A.L., Kinnee E.J., Tunno B.J., Manuck S.B., Gianaros P.J., Clougherty J.E. (2021). Long-Term Ambient Air Pollution Exposures and Circulating and Stimulated Inflammatory Mediators in a Cohort of Midlife Adults. Environ. Health Perspect..

[17] Tran H.M., Tsai F.-J., Lee Y.-L., Chang J.-H., Chang L.-T., Chang T.-Y., Chung K.F., Kuo H.-P., Lee K.-Y., Chuang K.-J. (2023). The impact of air pollution on respiratory diseases in an era of climate change: A review of the current evidence. Sci. Total Environ..

[18] Kogevinas M., Karachaliou M., Espinosa A., Aguilar R., Castaño-Vinyals G., Garcia-Aymerich J., Carreras A., Cortés B., Pleguezuelos V., Papantoniou K. (2023). Long-Term Exposure to Air Pollution and COVID-19 Vaccine Antibody Response in a General Population Cohort (COVICAT Study, Catalonia). Environ. Health Perspect..

[19] Zhang S., Chen S., Xiao G., Zhao M., Li J., Dong W., Hu J., Yuan T., Li Y., Liu L. (2022). The associations between air pollutant exposure and neutralizing antibody titers of an inactivated SARS-CoV-2 vaccine. Environ. Sci. Pollut. Res..

[20] Zeng Z., Ngai S., Wang Q., Liang W., Huo X. (2021). Early-life exposure to widespread environmental toxicants and children’s health risks: A focus on the post-vaccination antibody potency or immunoglobulin levels. Sci. Total Environ..

[21] Kinney P.L., Asante K.-P., Lee A.G., Ae-Ngibise K.A., Burkart K., Boamah-Kaali E., Twumasi M., Gyaase S., Quinn A., Oppong F.B. (2021). Prenatal and Postnatal Household Air Pollution Exposures and Pneumonia Risk: Evidence From the Ghana Randomized Air Pollution and Health Study. Chest.

[22] Franza L., Cianci R. (2021). Pollution, Inflammation, and Vaccines: A Complex Crosstalk. Int. J. Environ. Res. Public. Health.

[23] Fabris A.L., Nunes A.V., Schuch V., de Paula-Silva M., Rocha G., Nakaya H.I., Ho P.L., Silveira E.L.V., Farsky S.H.P. (2020). Hydroquinone exposure alters the morphology of lymphoid organs in vaccinated C57Bl/6 mice. Environ. Pollut..

[24] Bhat T.A., Kalathil S.G., Bogner P.N., Miller A., Lehmann P.V., Thatcher T.H., Phipps R.P., Sime P.J., Thanavala Y. (2018). Secondhand Smoke Induces Inflammation and Impairs Immunity to Respiratory Infections. J. Immunol..

[25] Liu K., Li S., Qian Z.M., Dharmage S.C., Bloom M.S., Heinrich J., Jalaludin B., Markevych I., Morawska L., Knibbs L.D. (2020). Benefits of influenza vaccination on the associations between ambient air pollution and allergic respiratory diseases in children and adolescents: New insights from the Seven Northeastern Cities study in China. Environ. Pollut..

[26] Liu K., Yang B.-Y., Guo Y., Bloom M.S., Dharmage S.C., Knibbs L.D., Heinrich J., Leskinen A., Lin S., Morawska L. (2020). The role of influenza vaccination in mitigating the adverse impact of ambient air pollution on lung function in children: New insights from the Seven Northeastern Cities Study in China. Environ. Res..

[27] Hong X., Morgenlander W.R., Nadeau K., Wang G., Frischmeyer-Guerrerio P.A., Pearson C., Adams W.G., Ji H., Larman H.B., Wang X. (2025). Maternal exposure to per- and polyfluoroalkyl substances and epitope level antibody response to vaccines against measles and rubella in children from the Boston birth cohort. Environ. Int..

[28] Semmes E.C., Chen J.L., Goswami R., Burt T.D., Permar S.R., Fouda G.G. (2020). Understanding Early-Life Adaptive Immunity to Guide Interventions for Pediatric Health. Front. Immunol..

[29] Wells A.C., Lotfipour S. (2023). Prenatal nicotine exposure during pregnancy results in adverse neurodevelopmental alterations and neurobehavioral deficits. Adv. Drug Alcohol. Res..

[30] Ruan Q., Jiang Y., Shi Y. (2024). Maternal smoking around birth and its influence on offspring allergic diseases: A mendelian randomization study. World Allergy Organ. J..

[31] Janbazacyabar H., van Bergenhenegouwen J., Garssen J., Leusink-Muis T., van Ark I., van Daal M.T., Folkerts G., Braber S. (2021). Prenatal and Postnatal Cigarette Smoke Exposure Is Associated With Increased Risk of Exacerbated Allergic Airway Immune Responses: A Preclinical Mouse Model. Front. Immunol..

[32] Dehghani A., Wang L., Garssen J., Styla E., Leusink-Muis T., van Ark I., Folkerts G., van Bergenhenegouwen J., Braber S. (2024). Synbiotics, a promising approach for alleviating exacerbated allergic airway immune responses in offspring of a preclinical murine pollution model. Environ. Toxicol. Pharmacol..

[33] Toutounchi N.S., Braber S., Van’t Land B., Thijssen S., Garssen J., Kraneveld A.D., Folkerts G., Hogenkamp A. (2021). Exposure to Deoxynivalenol During Pregnancy and Lactation Enhances Food Allergy and Reduces Vaccine Responsiveness in the Offspring in a Mouse Model. Front. Immunol..

[34] Xiao L., Leusink-Muis T., Kettelarij N., van Ark I., Blijenberg B., Hesen N.A., Stahl B., Overbeek S.A., Garssen J., Folkerts G. (2018). Human Milk Oligosaccharide 2’-Fucosyllactose Improves Innate and Adaptive Immunity in an Influenza-Specific Murine Vaccination Model. Front. Immunol..

[35] Oh S.S., Narver H.L. (2024). Mouse and Rat Anesthesia and Analgesia. Curr. Protoc..

[36] Tingskov Pedersen C.E., Eliasen A.U., Ketzel M., Brandt J., Loft S., Frohn L.M., Khan J., Brix S., Rasmussen M.A., Stokholm J. (2023). Prenatal exposure to ambient air pollution is associated with early life immune perturbations. J. Allergy Clin. Immunol..

[37] Yue H., Yan W., Ji X., Zhang Y., Li G., Sang N. (2018). Maternal exposure to NO2 enhances airway sensitivity to allergens in BALB/c mice through the JAK-STAT6 pathway. Chemosphere.

[38] Singh S.P., Gundavarapu S., Peña-Philippides J.C., Rir-Sima-ah J., Mishra N.C., Wilder J.A., Langley R.J., Smith K.R., Sopori M.L. (2011). Prenatal secondhand cigarette smoke promotes Th2 polarization and impairs goblet cell differentiation and airway mucus formation. J. Immunol..

[39] Strzelak A., Ratajczak A., Adamiec A., Feleszko W. (2018). Tobacco Smoke Induces and Alters Immune Responses in the Lung Triggering Inflammation, Allergy, Asthma and Other Lung Diseases: A Mechanistic Review. Int. J. Environ. Res. Public. Health.

[40] Shibuya M., Aoshi T., Kuroda E., Yoshioka Y. (2020). Murine Cross-Reactive Nonneutralizing Polyclonal IgG1 Antibodies Induced by Influenza Vaccine Inhibit the Cross-Protective Effect of IgG2 against Heterologous Virus in Mice. J. Virol..

[41] Firacative C., Gressler A.E., Schubert K., Schulze B., Müller U., Brombacher F., von Bergen M., Alber G. (2018). Identification of T helper (Th)1- and Th2-associated antigens of Cryptococcus neoformans in a murine model of pulmonary infection. Sci. Rep..

[42] Restori K.H., Srinivasa B.T., Ward B.J., Fixman E.D. (2018). Neonatal Immunity, Respiratory Virus Infections, and the Development of Asthma. Front. Immunol..

[43] Janbazacyabar H., van Daal M., Leusink-Muis T., van Ark I., Garssen J., Folkerts G., van Bergenhenegouwen J., Braber S. (2021). The Effects of Maternal Smoking on Pregnancy and Offspring: Possible Role for EGF?. Front. Cell Dev. Biol..

[44] Kim Y.-J., Choi M.J., Bak D.-H., Lee B.C., Ko E.J., Ahn G.R., Ahn S.W., Kim M.J., Na J., Kim B.J. (2018). Topical administration of EGF suppresses immune response and protects skin barrier in DNCB-induced atopic dermatitis in NC/Nga mice. Sci. Rep..

[45] CDC Health Effects of Cigarettes: Reproductive Health. Atlanta, GA, USA. 2025. https://www.cdc.gov/tobacco/about/cigarettes-and-reproductive-health.html.

[46] Hammer B. (2020). Parental Smoking Behavior: Cellular and Molecular Consequences for Murine Offspring.

[47] Hjálmsdóttir Á., Hasler F., Waeckerle-Men Y., Duda A., López-Deber M.P., Pihlgren M., Vukicevic M., Kündig T.M., Johansen P. (2024). T cell independent antibody responses with class switch and memory using peptides anchored on liposomes. npj Vaccines.

[48] Raval F.M., Mishra R., Garcea R.L., Welsh R.M., Szomolanyi-Tsuda E. (2013). Long-lasting T cell-independent IgG responses require MyD88-mediated pathways and are maintained by high levels of virus persistence. mBio.

[49] de Vinuesa C.G., Cook M.C., Ball J., Drew M., Sunners Y., Cascalho M., Wabl M., Klaus G.G., MacLennan I.C. (2000). Germinal centers without T cells. J. Exp. Med..

[50] Chen L., Bennett E., Wheeler A.J., Lyons A.B., Woods G.M., Johnston F., Zosky G.R. (2018). Maternal exposure to particulate matter alters early post-natal lung function and immune cell development. Environ. Res..

[51] Hong X., Liu C., Chen X., Song Y., Wang Q., Wang P., Hu D. (2013). Maternal exposure to airborne particulate matter causes postnatal immunological dysfunction in mice offspring. Toxicology.

[52] Huang K.L., Liu S.Y., Chou C.C., Lee Y.H., Cheng T.J. (2017). The effect of size-segregated ambient particulate matter on Th1/Th2-like immune responses in mice. PLoS ONE.

[53] Allouche J., Cremoni M., Brglez V., Graça D., Benzaken S., Zorzi K., Fernandez C., Esnault V., Levraut M., Oppo S. (2022). Air pollution exposure induces a decrease in type II interferon response: A paired cohort study. EBioMedicine.

[54] Lu C., Zhang Y., Li B., Zhao Z., Huang C., Zhang X., Qian H., Wang J., Liu W., Sun Y. (2022). Interaction effect of prenatal and postnatal exposure to ambient air pollution and temperature on childhood asthma. Environ. Int..

[55] Wright R.J., Hsu H.L., Chiu Y.M., Coull B.A., Simon M.C., Hudda N., Schwartz J., Kloog I., Durant J.L. (2021). Prenatal Ambient Ultrafine Particle Exposure and Childhood Asthma in the Northeastern United States. Am. J. Respir. Crit. Care Med..

[56] Mountford A.P., Fisher A., Wilson R.A. (1994). The profile of IgG1 and IgG2a antibody responses in mice exposed to Schistosoma mansoni. Parasite Immunol..

[57] Moran T.M., Park H., Fernandez-Sesma A., Schulman J.L. (1999). Th2 responses to inactivated influenza virus can Be converted to Th1 responses and facilitate recovery from heterosubtypic virus infection. J. Infect. Dis..

[58] Bungener L., Geeraedts F., Ter Veer W., Medema J., Wilschut J., Huckriede A. (2008). Alum boosts TH2-type antibody responses to whole-inactivated virus influenza vaccine in mice but does not confer superior protection. Vaccine.

[59] Crawford L., Halperin S.A., Dzierlenga M.W., Skidmore B., Linakis M.W., Nakagawa S., Longnecker M.P. (2023). Systematic review and meta-analysis of epidemiologic data on vaccine response in relation to exposure to five principal perfluoroalkyl substances. Environ. Int..

[60] Looker C., Luster M.I., Calafat A.M., Johnson V.J., Burleson G.R., Burleson F.G., Fletcher T. (2013). Influenza Vaccine Response in Adults Exposed to Perfluorooctanoate and Perfluorooctanesulfonate. Toxicol. Sci..

