# Supplementary material for: Maternal Cigarette Smoke Exposure Does Not Impair Influenza Vaccine Responsiveness in Murine Offspring
Source: Vaccines (Basel). 2025 Oct 16;13(10):1058. doi: 10.3390/vaccines13101058 (PMC12568188; doi:10.3390/vaccines13101058)
Supplement: Supplementary file 1 [file vaccines-13-01058-s001.zip › vaccines-3775023-supplementary.pdf]

# Supplementary data

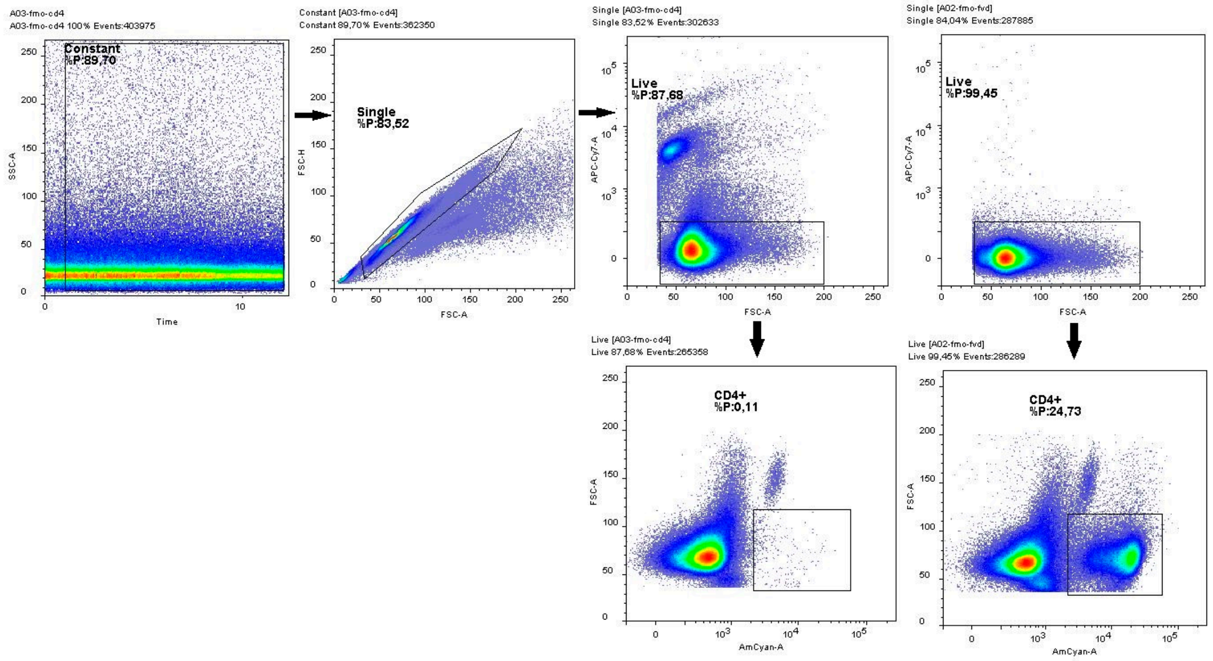


**Supplementary Figure S1.** Gating strategy used for FACS analysis of T cells in solenocytes from Influenza-vaccinated offspring born to air-exposed and CS-exposed dams, performed using FlowLogic software.

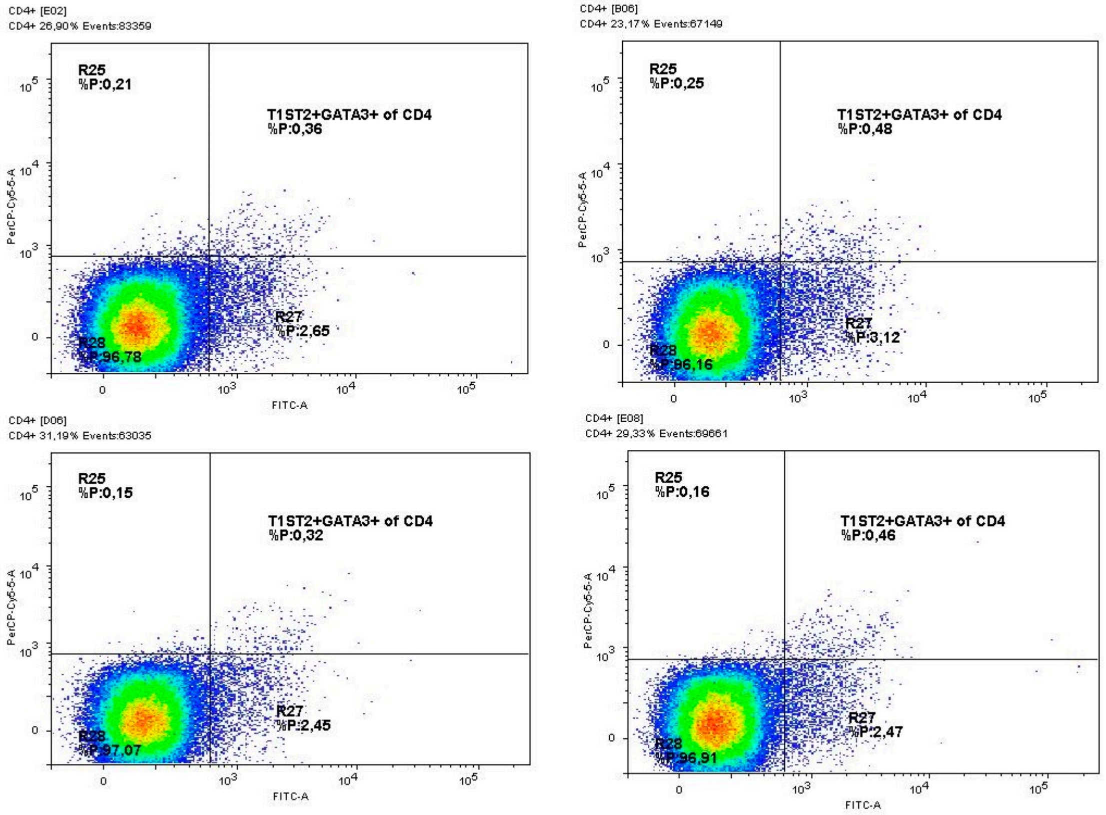

**Supplementary Figure S2.** Gating strategy used for FACS analysis of Th2 cells (% T1ST2+, GATA3+, CD4+) in solenocytes from Influenza-vaccinated offspring in offspring born to air-exposed and CS-exposed dams, performed using FlowLogic software.
